# Housing, Affordability, and Community-Based Aging

**DOI:** 10.1093/geroni/igaa057.2415

**Published:** 2020-12-16

**Authors:** Samara Scheckler

## Abstract

The house acts as both an environment of care and a vehicle to financially potentiate long-term community-based support. While housing can empower a diverse set of options for a person-centered aging process, inadequate housing can also impede healthy aging in the community. This symposium teases out the nodes where housing acts to benefit or limit safe community-based aging. The first paper in this symposium, Homeownership Among Older Adults, describes typologies of older adult homeownership and sensitively highlights trends, disparities and important considerations of homeownership in later life. The next two papers take these older adults and explores situations where their housing acts as an asset or as a burden. Identifying Cost Burdened Older Adults acknowledges that housing cost burdens look different for older adults than younger cohorts. A more precise definition of older adult housing cost burden is proposed to help researchers and policymakers better synthesize the complex relationships between older adult housing and their long-term care decisions. The Long-Term Care Financing Challenge then explores the role of home equity in expanding the community-based long-term care choice set for older adults. This paper demonstrates benefits (both realized and unrealized) in home equity and suggests policy implications moving forward. Finally, Cardiometabolic Risk Among Older Renters and Homeowners disentangles the relationship between housing and health by demonstrating health disparities that are associated with housing tenure, conditions and affordability. Taken together, this symposium explores the complex and multidirectional relationships between housing, long-term care and older adult health.

